# Editorial: Nutrition and management of animals we keep as companions, volume II

**DOI:** 10.3389/fvets.2023.1348594

**Published:** 2023-12-19

**Authors:** Luciano Trevizan, Anna K. Shoveller, Ananda P. Félix

**Affiliations:** ^1^Department of Animal Science, Universidade Federal do Rio Grande do Sul, Porto Alegre, Rio Grande do Sul, Brazil; ^2^Department of Animal Biosciences, University of Guelph, Guelph, ON, Canada; ^3^Department of Animal Science, Universidade Federal do Paraná, Curitiba, Paraná, Brazil

**Keywords:** digestibility trials, ingredients, lipid metabolism, pet food processing, camelina oil

## 1 Summary and commentary

Addressing the conflict between global warming and consumption of food by agricultural, companion, and exotic animals and humans requires a holistic approach that considers sustainable agricultural practices, efficient ingredient and product supply chains, reduced food waste, consideration of upcycling of food waste, and changes in consumption patterns. Invention and innovation in technology, policy interventions, and knowledge translation to individual consumer choices all play a role in finding solutions to mitigate the environmental impact of food production and consumption. The profound challenges that we face oblige us to find solutions that optimize animal, human, and environmental health.

In the nutrition of dogs and cats, animal sources of ingredients have been postulated as more complete ingredients, considering the nutrient requirements of these animals. However, the number of pet owners willing to replace animal sources with plant-based ingredients is growing. Pet owners believe they can potentially reduce their pets' environmental impact when transitioning to plant-based pet foods. In fact, it makes sense, since animal sources come from another trophic level, and the environmental cost is higher than that of producing vegetable ingredients. However, many pet foods use animal by-products, which do not involve directly raising livestock. Further to this, plant-based ingredients do not deliver all the nutrients that dogs and cats require. Thus, in this edition, Yoosefzadeh-Najafabadi et al. discuss how plants could be selected in a breeding program to get high-quality cultivars to meet the future demands of ingredients in feed formulations intended for pets. The authors addressed that with the improvement of the nutritional quality of plant-based feedstuffs, it may be possible to reduce the number of ingredients used to formulate complete diets for pets.

In this context, Morris E. et al. included vegetable protein in diets for cats, replacing hydrolyzed chicken liver and heart with rice protein concentrate at 0, 7, 14, and 28% of the diet. A linear improvement in the digestibility of dry matter and energy was observed followed by a linear improvement in true protein and carbohydrate digestibility as well. Against all expectations, food consumption was greater and fecal consistency was improved in cats fed the rice concentrate protein, making it an excellent ingredient to include in cat diets.

On the other side of society, there are pet owners interested in going against processed food, looking to feed dogs and cats with raw food, which they call “natural” foods. However, the risk of feeding companion animals with raw food has been declared not just for the animals but also for humans living with them. According to Kiprotich and Aldrich, some strategies can be used to minimize the risk of pathogens in raw diets, such as using non-thermal processing, high-pressure pasteurization, or using Generally Recognized as Safe (GRAS) food additives approved such as organic acids, essential oils, and bacteriophages can be considered as effective methods. The association of different additives and their combination with methods holds promise for controlling pathogenic microbes in raw food and ensuring the safety of these foods for both animals and the humans they share their households with.

Hemida et al. suggested that raw and extruded diets can have effects on epigenetics related to otitis in dogs. After evaluating longitudinal data reported in mothers and puppies, they concluded that dogs fed an ultra-processed carbohydrate-based diet (UPCD, dry) - 75% or more during pregnancy or in the growing phase of puppies up to6 months - were associated with otitis in later life. Puppies fed a non-processed meat-based diet (NPMD, raw) - at least 25% of the total diet - were associated with an increase in the incidence of otitis. Also, exposure to sunlight for more than 1 h a day and dogs raised on a dirt floor had a lower risk of developing otitis suggesting that the environment we raise pets in also predicts the risk of otitis.

Homemade diets may be a risk to dogs' and cats' health when not well-balanced. However, well-adjusted homemade diets for specific animals can benefit health. Silva et al. reported a case of ALT likely increased by excessive hepatic copper of a dog fed a kibble diet. Despite the concentrations being under normal levels, it is recognizable that sensitive dogs can be affected by copper concentrations that are relatively low. Using a homemade diet with moderately low copper was enough to maintain the dog with a low concentration of serum ALT.

The association between grain-free diets and dilated cardiomyopathy (DCM) is still under investigation after 2018 when the US Food and Drug Administration reported a potential link between these two factors. Bokshowan et al. investigated if the replacement of rice by pea, or rice diet plus 1% raffinose, is related to changes in the bioavailability of taurine, sulfur amino acids, excretion of bile salts, and some changes in the dynamics of the heart that could be related to the development of DCM after 5 weeks of feeding. Taking all results together, they did not find a clear relationship between pea or oligosaccharide-containing diets. Instead, a control diet (commercial dental diet) with no oligosaccharides detectable, but with a higher level of insoluble fiber, produced changes in plasma N-terminal pro-brain natriuretic peptide (NT-proBNP), one of the indicators of the development of DCM. In fact, some nutritional traits common in grain-free diets, such as gluten-free and low-glycemic starch sources, may present beneficial physiological effects in specific cases. Baptista da Silva et al. reported a case of a German Spitz with epileptic seizures controlled by a gluten-free/hydrolyzed protein diet. Also, Vastolo et al. tested two grain-free diets (sweet potato vs. pea starch) against a control diet (spelt + oats) to look at the postprandial glycemia in dogs fed different starch sources. The diet containing pea starch had the smallest postprandial glucose and insulin area under the curve and the lowest serum concentration of fructosamine, followed by the diet containing sweet potato.

Different starch sources can release glucose differently in the intestine, presenting an impact on glycemia and the net disposal of glucose in peripheral tissues. The dynamics of glucose digestion and metabolism need further investigation. Dogs and cats with diabetes need special formulations, and functional ingredients are fundamental to producing therapeutic diets. Recently, Corbee et al., looking for another way to control glycemia in diabetic patients, reviewed the fibroblast growth factor-21 (FGF21) analogs as a possible option to help in the treatment of diabetes. FGF21 is produced normally in the body and is greater in obese and diabetic patients. In experimental models, FGF21 seems to improve hepatic glucose metabolism, enhance serum insulin concentrations, lower blood glucose, and stimulate β-oxidation of the fatty acids. Curiously, at increasing concentrations of FGF21, obese mice did not fully respond anymore, leading the authors to believe that FGF21 could be harmful to the body and metabolism, or obese patients could become resistant to FGF21. The analogs of FGF21 have not been tested in dogs, but the combination with diets and exercise would be an efficient proposal for weight loss and glycemia control.

Food processing has been the focus of research for a long time as a way to produce and store food safely. Dainton, Molnar et al. investigated the effects of the canned food container size and type (flexible and semi-rigid and rigid) on thermal processing and its effect on B vitamins. They observed that the more flexible the containers are, the faster they reach the target lethality (time vs. temperature) during retort processing. Thiamine and riboflavin were shown to be unstable during retort processing, independent of the container type evaluated. The other vitamins remained stable. However, the authors drew attention to the processing losses, which must be greater once the target lethality was reached in this study, and supplementation is needed to guarantee the required amount of vitamins. Also, Dainton, White et al. showed that supplementing vitamin premix and yeast could improve the levels of thiamine in canned food. After 6 months, diets containing yeast were associated with a greater thiamin concentration, suggesting that thiamine from yeast is more resistant to storage.

Vitamins and other nutrients are adjusted to the content of energy in the diet to secure adequate ingestion of essential nutrients. The lack of precision in estimating metabolizable energy could result in malnutrition or add excessive nutrients, which is environmentally wasteful. Jewell and Jackson reviewed the equations to predict energy in dry and wet food for dogs and cats using a large amount of data and compared them to the energy density prediction values of the modified Atwater factors, the NRC (crude fiber), and the Hall equations. New equations were proposed, tested, and compared to the others. Despite the errors associated with the equations, a new version reduced the difference between the measured and estimated metabolizable energy, and a better predictor of food consumption per metabolic body weight was reached for dogs and cats, helping to adjust nutrients to the content of energy and avoiding over formulation and waste of nutrients.

Lipids are an important energy source for dogs and cats. Also, fatty acids are functional molecules and play a role as structural compounds in the cells. Jackson and Jewell conducted a study on long-chain fatty acids using fish oil (FO) as a source of EPA (20:5n3) and DHA (22:6n3) and medium-chain fatty acids oil (MCT - 8% caproate, 6:0; 51.4% caprylate, C8:0; 39.1% caprate, C10:0; < 0.1% laurate, C12:0; and < 0.01% of other fatty acids). They fed dogs for 28 days, and serum metabolomics was performed. The inclusion of FO decreased levels of triglycerides and total cholesterol, whether isolated or in association with MCT. Overall, the metabolites found showed that MCT largely led to changes in serum lipids associated with energy metabolism, while FO consumption produced changes dominated by structural-type lipids, confirming the competition already known between n-3 and n-6 fatty acids on phospholipids according to their inclusion in the diet. Furthermore, a long list of lipids was found and explored in the study, most of them were linked to the immune system and signaling factors, and part of them responded to the FO diet. Richards et al. also investigated the effects of concentrated vegetable sources of n-3 in dogs. Camelina oil, the second richest ALA oil, was compared with flaxseed and canola oil, searching for changes in inflammatory and oxidative markers, and coat quality in adult dogs. Camelina oil improved the coat, as did all other sources of oil containing great concentrations of linolenic acid (18:2n-3). However, inflammatory and oxidative markers remained stable across the diets and for 16 weeks of supplementation; however, all dogs were healthy and without any disorders mediated by inflammation.

The lipidic and energetic metabolism of the organism depends on some nitrogenous compounds, such as creatine, choline, and carnitine to work properly. For example, phosphocreatine is readily used for ATP regeneration during initial high caloric demand, while choline and carnitine are important for phospholipid synthesis and hepatic export and β-oxidation of long-chain fatty acids, respectively. Banton et al. showed that feeding healthy dogs with a diet supplemented with a combination of creatine, carnitine, and choline (CCC diet) resulted in the elevation of plasma creatine concentration more than diets supplied with methionine or taurine. Plasma creatine remained elevated up to 6 h after the meal. As a consequence, concentrations of plasma creatinine were elevated from 1 to 3 h after the meal in dogs fed the CCC diet. The fact that plasma creatinine can be affected by dietary creatine in the diet warns of potentially misleading results in healthy patients when creatinine is used as a diagnostic. Choline and carnitine were also tested by Rankovic et al. in lean and obese cats. Choline supplied in the diet resulted in lower food intake in cats, but no changes in body weight, body condition score, and body composition were observed. Lipid metabolism was influenced by choline, and greater total cholesterol and its fractions were increased in plasma. It seems that choline improves the mobilization of lipids. Choline or carnitine seemed not to play any influence over obese and lean cats, although obese cats showed more serum triglycerides, alkaline phosphatase, VLDL, and HDL-C than lean cats.

Besides the relevance of studying the functional role of nutrients in dogs and cats, the evaluation of diet digestibility is crucial during pet food development. Research and development are one of the industrial sections that require special attention. Improving the digestibility of diets has an impact on nutrient availability and fecal consistency. Jadhav et al., adding a blend of enzymes to commercial diets *in vitro*, observed greater digestibility of nutrients and energy, protein molecular weight reduction, and an increase in antioxidant capacity, providing an effective strategy to enhance nutrient digestibility. Classically, digestibility is performed *in vivo* with trained animals following AAFCO and FEDIAF protocols. However, Bos et al. evaluated an in-home protocol of digestibility in cats using the marker method (TiO_2_). They fed cats for 8 days, and feces were collected and analyzed on a daily basis. A steady state was reached in digestibility parameters within 2 days of adaptation to the diet suggesting digestibility studies do not require feeding animals for longer periods of time. Also, after adaptation, 1 day of fecal collection was accurate to define digestibility at home, and using 3 days improved accuracy based on the irregular defecation pattern of the cats. Depending on the nutrient class, the number of cats ranged between 6 and 12, assuming acceptable errors. However, the authors declare that a comparison between in-home and in-lab tests would be interesting to control the sources of variation with in-home digestibility tests. Adoption of in-home digestibility protocols would help to avoid the reduce the use of laboratory dogs and cats in these research protocols.

Complementary to diet digestibility evaluation, analysis of dietary salt content and effects on urine production and supersaturation are very important, especially to cats. The relative urine supersaturation (RSS) can be calculated by software (EQUIL2). Anthony et al. used EQUIL2 to calculate the RSS and compared it with the RSS calculated in a new version of the software (EQUIL-HL21), and both were considered satisfactory for data from dogs and cats. Also, Morris E. M. et al. demonstrated that the EQUIL-HL21 program can accurately detect expected differences between foods formulated for urinary and non-urinary indications for cats. Regression models revealed that pH, magnesium, ammonium, citrate, chloride, calcium, phosphorus, and sulfate are the main urinary analytes that contribute to the predicted RSS values for struvite and calcium oxalate crystal formation. Urinary foods produced lower urinary pH, ammonium, potassium, phosphorus, magnesium, oxalate, citrate, and sulfate concentrations. The greater amount of sodium and chloride in urinary diets improves their excretion and increased volume of urine both which helps with dilution and lowers the risk for struvite formation.

Finally, in this second edition of “*Nutrition and management of animals we keep as companions*,” we had the opportunity to review a diverse array of studies employing various approaches, all aimed at addressing questions related to pet nutrition. Ingredients, nutrients, epigenetic effects, absorption, and metabolism were evaluated in both healthy and ill patients, providing a comprehensive perspective to integrate information and determine optimal feeding practices for the health and longevity of dogs and cats.

## Author contributions

LT: Conceptualization, Writing—original draft, Writing—review & editing. AS: Conceptualization, Writing—review & editing. AF: Conceptualization, Writing—review & editing.

